# CSI-OMIM - Clinical Synopsis Search in OMIM

**DOI:** 10.1186/1471-2105-12-65

**Published:** 2011-03-01

**Authors:** Raphael Cohen, Avitan Gefen, Michael Elhadad, Ohad S Birk

**Affiliations:** 1 The Morris Kahn Laboratory of Human Genetics, National Institute for Biotechnology in the Negev (NIBN), Ben-Gurion University, Beer-Sheva, Israel; 2 Department of Computer Science, Ben-Gurion University, Beer-Sheva, Israel

## Abstract

**Background:**

The OMIM database is a tool used daily by geneticists. Syndrome pages include a Clinical Synopsis section containing a list of known phenotypes comprising a clinical syndrome. The phenotypes are in free text and different phrases are often used to describe the same phenotype, the differences originating in spelling variations or typing errors, varying sentence structures and terminological variants.

These variations hinder searching for syndromes or using the large amount of phenotypic information for research purposes. In addition, negation forms also create false positives when searching the textual description of phenotypes and induce noise in text mining applications.

**Description:**

Our method allows efficient and complete search of OMIM phenotypes as well as improved data-mining of the OMIM phenome. Applying natural language processing, each phrase is tagged with additional semantic information using UMLS and MESH. Using a grammar based method, annotated phrases are clustered into groups denoting similar phenotypes. These groups of synonymous expressions enable precise search, as query terms can be matched with the many variations that appear in OMIM, while avoiding over-matching expressions that include the query term in a negative context. On the basis of these clusters, we computed pair-wise similarity among syndromes in OMIM. Using this new similarity measure, we identified 79,770 new connections between syndromes, an average of 16 new connections per syndrome. Our project is Web-based and available at http://fohs.bgu.ac.il/s2g/csiomim

**Conclusions:**

The resulting enhanced search functionality provides clinicians with an efficient tool for diagnosis. This search application is also used for finding similar syndromes for the candidate gene prioritization tool S2G.

The enhanced OMIM database we produced can be further used for bioinformatics purposes such as linking phenotypes and genes based on syndrome similarities and the known genes in Morbidmap.

## Background

### Problem Description

Mendelian syndromes are monogenic hereditary diseases, each manifesting as a combination of clinical signs and symptoms (phenotypes). Syndromes can differ from each other by as few as one or two phenotypes. Not all patients display all of the known phenotypes, even if they all have the same syndrome; for example, for the Cystic Fibrosis syndrome, only 10% of the patients display the "*meconium ileus*" phenotype [OMIM 219700]. Genetic Mendelian syndromes are specifically associated to a gene or group of genes.

The most important curated, comprehensive, reliable, and updated source of information in human genetics is the Online Mendelian Inheritance in Man (OMIM) databank [1], which consists of some 17,000 detailed entries on human genes and genetic disorders including 4,802 syndromes with a Clinical Synopsis section (described below). This database is routinely used by clinicians to diagnose patients and is also used widely for data mining purposes [2-4].

Each OMIM entry contains free text descriptions of: genetic loci, inheritance patterns, allelic variants, biochemical and clinical features, and molecular and population genetics. Most OMIM syndrome entries also include a Clinical Synopsis (CS) section in structured text that outlines signs and symptoms (phenotypic features) accompanying the disease. The phenotypes are presented within one or two levels of contextual information: the first describing the heading or body system (such as "Head", "Heme" or "Labs") and an optional subheading (for example "Eyes" may come under "Head and Neck" and "Airways" comes under "Respiratory" in [OMIM 272800]).

Two problems with searching and mining OMIM are that the same phenotype can be described using very different phrases and that negations are used widely to indicate lack of a phenotype.

The similarity between two syndromes can be calculated using the number of shared phenotype phrases. Regrettably, information in the CS sections is not represented in a uniform manner. No controlled vocabulary is used for phenotype and location names. Typing errors and synonyms for the same entity, as well as different nomenclature for overlapping concepts, are observed in large numbers: "*trunk ataxia*" vs. "*truncal ataxia*", "*hypoplastic radius*" vs. "*hypoplastic radii*", "*brachymetacarpalia*" vs. "*brachymetacarpals*", "*cystic renal dysplasia*" vs. "*renal dysplasia cystic*" vs. "*renal cystic dysplasia*" and many more.

Negation phrases are phenotype phrases describing symptoms the patients do not have, therefore adding mistakes to search results. For example, when searching for "*loss of vision*", we are not interested in retrieving syndromes containing the description "*no significant loss of vision*". This further confounds calculating the similarity between two syndromes since sharing a negation phrase does not mean the syndromes are similar.

### Previous Work

OMIM has already been used as a data source for data mining in previous research. Freudenberg *et al*. [5] manually processed syndrome data in OMIM and collected 5 fields of information for each syndrome (episodic, etiology, tissue, onset and inheritance). The syndrome information was used to find similarity between syndromes for prediction of gene involvement in diseases. Their work was performed manually and the results capture only limited aspects of the syndrome descriptions.

In GFINDer [4], OMIM syndromes are used for enrichment of gene lists obtained with microarray experiments.

Cantor and Lussier [2] used phenotype headings for clustering syndromes. Only the headings and sub-headings were used due to the problematic nature of the free-form clinical synopsis information in OMIM. In this work, we address this limitation by analyzing the free-form text of the phenotypes.

In 2006, Driel *et al*. [6] have created an algorithm for automatically mapping the OMIM descriptions to MESH (Medical Subject Headings) [7] terms. This mapping also aims at normalizing the free-form OMIM phenotype descriptions. The objective of this processing was similar to ours: to discover connections between phenotypes and syndromes. The MESH controlled vocabulary does not contain all the concepts appearing in OMIM. As a result, this approach leads to significant loss of data in OMIM, which has no MESH terms associated with it, as well as some of the phenotype context.

Unified Medical Language System (UMLS) [8] is a larger medical language resource provided by the NLM. It consists of a collection of medical vocabularies. UMLS MetaMap [9] maps texts into UMLS concepts. Lage *et al*. [3] used UMLS MetaMap [9] for mapping syndromes to UMLS terms (instead of MESH). Their aim was to use syndrome similarity in order to predict disease gene association.

This approach produces limited success, as many of the associated syndromes found were simply variants of the same syndrome ("*Waardenburg syndrome type IIA*" and "*Waardenburg syndrome type IIB*") or poor quality association. For example, "*Tuberous sclerosis (TS)*" and "*Chordoma*" were found as similar in their analysis. This association is based on reports of both diseases found in 3 patients. This association was not described in the Clinical Synopsis, as it is likely a random association due to the relative large number of TS patients. Further comparison to this work is not possible as the full results of the OMIM analysis by Lage *et al*. [3] were not published.

The methods of Driel *et al*. (2006) [6] and Lage *et al*. (2007) [3] both ignore the context in which terms occur in the OMIM entries and do not detect negative contexts around term entities (negation detection such as NegEx [10]). Neither method provides a practical software system for searching OMIM.

UMLS MetaMap performance in extracting biomedical terms was examined in a few articles. Pratt and Yetisgen-Yildiz [11] compared MetaMap to three human experts and found precision of 55% and recall of 93%. Chapman *et al*. [12] examined MetaMap in the domain of respiratory findings in emergency department reports and reported a precision of 56% and recall of 72% for clinical terms. Meystre and Haug [13] examined results using MetaMap with all of UMLS or when defining a subset of UMLS relevant to 80 medical conditions they were interested in. The default data set produced precision of 76% percent and recall of 74% while the customized subset produced similar precision (69%) with an improved recall of 90%.

Another system for identifying UMLS terms in a document is BioMedLEE by Lussier and Friedman [14], with reported precision of 89% and recall of 77%. Unfortunately BioMedLEE was not available for comparison with our work.

Robinson *et al*. (2008) [15] manually created a database of phenotypes and syndrome relations from OMIM, reported in 2010 [16] to contain more than 9,500 phenotype phrases built into ontology. Our method is automatic and captures more phenotype phrases (almost 3 times as many phrases) in a more superficial manner (as manual clustering is more precise).

S2G (Syndrome to Gene) [17], is a tool for finding candidate genes for hereditary syndromes in suspect loci. S2G is comprised of two parts: one for ranking genes in a locus in comparison to a known gene causing the syndrome, the other, for syndromes with no known genes uses our search application for choosing a known gene causing the most phenotypically similar syndrome.

### Our Approach

Recognizing which phenotype phrases denote similar phenotypes and recognizing negation phrases helps searching (we can match the query term to all the variations under which it appears in the database) and provides critical input to further data-mining of the rich information stored in OMIM.

Our objective in this work is to pre-process the natural language descriptions that appear in OMIM to identify synonymous phrases denoting the same phenotype. For example, we want to identify that "*unilateral kidney agenesis*" and "*unilateral renal agenesis*" both denote the same phenotype. Once we have identified such clusters of synonymous phrases, we can normalize the natural language descriptions in OMIM: we can recognize that two syndromes include the same phenotype even though their natural language descriptions are different.

In our approach, we also attempt to use MetaMap and the alignment it can generate between the raw OMIM phrases and UMLS terms (as done by [3]). But this data provides only partial categorization and we found it not to be robust. We do not assume a priori that OMIM terms will be found in an existing ontology or controlled category (such as MESH or UMLS). In contrast, we identify clusters of similar phrases (and combine several clues to determine similarity). Our approach is close, in terms of methodology, to the WordNet (a lexical database for the English language) approach to thesaurus construction: WordNet does not assume a priori that a sense hierarchy exists. Instead, it identifies classes of words that share a similar sense by grouping them in the same class. These classes are called synsets. Similarly, in our method, the clusters of similar phenotype phrases emerge through the computation of pair-wise similarity. We find in our experiment that this method is more robust to the noisy data observed in a large corpus such as OMIM, which evolves over a long period of time and is maintained by a variety of experts. Precision found in predicted synonymous phrase pairs was 93.5% (500 pairs of phrases were sampled randomly from the resulting phrase clusters and evaluated by a geneticist as similar or non-similar). 10% of the phrases were recognized as negations with a precision of 89% with most false positive being ambiguous.

Using the discovered phenotype clusters, we have created a Web application for searching OMIM, called CSI-OMIM, which provides much more efficient search than the original OMIM site. CSI-OMIM supports incremental search in the following manner: the clinician enters free text description for a phenotype. The application displays a list of matching phenotype clusters found by partial string matching to the query. The user can select the best matching clusters and continue searching for more phenotypes. At any time, the user can also search for the most similar syndromes matching the list of selected phenotype clusters.

CSI-OMIM obtains high recall in the search for phenotypes, because it captures the wide variability found in the OMIM free-text descriptions. It also has improved precision (with respect to the original OMIM search engine) because it avoids retrieving phrases in negative contexts. Finally, the matching from phenotype clusters to syndrome is highly effective, because it uses the reliable syndrome-similarity measure (cosine similarity over the all the phenotypes) computed over the phenotype clusters.

In the rest of the paper, we present our method to compute clusters of similar phenotype phrases from the various OMIM free-text descriptions, using natural language processing methods. We then present an evaluation of the quality of the acquired phenotype clusters and of the syndrome similarity measure induced from the clusters of similar phenotype phrases.

## Construction and content

### Data Collection

To prepare our analysis, we extracted and formatted data out of the OMIM database according to the following method. All Clinical Synopsis (CS) entries of the syndromes were used to build a database of syndrome phenotypes; the OMIM text data is obtained from the NCBI site (see details in Additional File 1) and the system is updated every three months using an automatic process. The updated data, OMIM data used for the analysis in this paper was obtain on October 13, 2010, is automatically processed using the process described below (parsing using a Context Free Grammar and annotating semantic types using MetaMap. We constructed manually the CFG grammar used to parse the text, but the parsing is performed automatically on each update).

### Defining Phenotype Areas

The contextual information of a phrase (its heading and sub-heading) is crucial for phenotype phrase comparison. Only phrases with similar context should be compared to avoid false positive results.

The list of headings and sub- headings is not strictly maintained in OMIM. The same heading appears in different syndromes under slightly different names. Phenotypes can also appear directly below headings, without a sub-heading refinement. This variability makes it difficult to systematically exploit the contextual information provided by the heading/sub-heading classification.

To address this lack of consistency, we manually defined 26 areas. We selected the areas to best describe the large and overlapping number of domains and sub domains. For example: the "Head and Neck" heading includes both the "Head" and "Neck" domains. "Head and Neck" and "Head" are both included in area 17 - "Head", area 16 - "Neck" includes "Neck" and "Head and Neck".Table 1 in the Results section shows the list of areas we constructed.

**Table 1:** Areas of phenotypes identified

Area Name	Area#	#Distinct phrases	#Clusters identified	Avg Similar Phrases Cluster Size	% Phrases clustered in area
Syndrome names	1	4,801	278	3.45	19.9

Abdomen/gi	2	707	33	2.3	10.1

Respiratory	3	546	39	2.17	15.6

Gu/renal	4	985	44	2.25	10.0

Gu/genitalia	5	975	44	2.25	10.0

Cardiovascular	6	808	38	2.13	10.0

Muscle	7	1143	68	2.98	17.8

Endo	8	344	16	2.25	10.4

Neuro	9	4.721	247	2.68	14.0

Oncology	10	891	21	2.62	6.17

Heme	11	576	16	2.75	7.6

Immune	12	542	19	2.05	7.2

Eyes	13	2,265	152	2.5	16.8

Face	14	3,927	283	2.56	18.4

Teeth	15	3,542	258	2.48	18.1

Neck	16	3,490	252	2.49	17.9

Head	17	4,196	281	2.51	16.8

Limb	18	4,522	283	3.15	19.7

Skel	19	3,936	247	3.02	18.9

Chest	20	4,307	273	3.03	19.2

Growth	21	497	37	4.24	31.6

Nails	22	1,617	106	2.41	15.8

Skin	23	2,098	140	2.42	16.2

Hair	24	1,701	116	2.41	16.5

Lab	25	3,553	106	2.9	8.6

Misc	26	4,021	Was not clustered		

### Similarity Computation

#### Combining Cues to Compare Phrases

The task we now approach is the following: given a set of phrases within a single area, how can we determine the level of similarity between any two phrases. For example, how do we recognize that "*flaring of the iliac wings*" and "*flared iliac wing*" are similar, but "*flared metaphyses*" and "*flared iliac wing*" are not.

**Figure 1:**
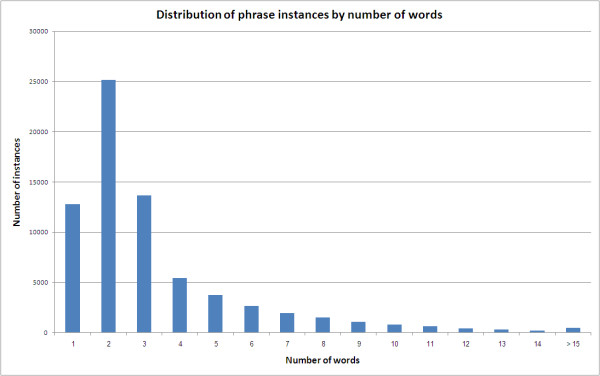
**Distribution of phrase instances by number of words**. Distribution of length of Noun Phrases in OMIM Clinical Synopsis.

The phrases observed in the OMIM dataset are all noun phrases. On average, they include 3.9 words; with a maximum of 64 words for the most complex phrase (found in syndrome 255125 in OMIM). Syntactically, the phrases exhibit structures such as Noun, Adjective Noun, and a few Prepositional Phrases. Figure 1 illustrates the distribution of the length of the Noun Phrases observed in the OMIM Clinical Synopses. Most of the phrases (80% out of 67,470 phrases) include between 2 and 6 words (see Figure 1).

Our intuition when comparing noun phrases is to combine several sources of knowledge about the phrases to decide on their similarity. First, because the vocabulary used in OMIM is complex and not fully consistent (spelling variations and usage of synonyms are both common), we attempt to align each word with a known controlled vocabulary (we use UMLS and MetaMap). Second, because phrases exhibit complex syntactic structures (2 to 6 words), we attempt to parse the phrases into trees with explicit modification relations. Third, because there can be slight spelling variations in the phrases, we also use a simple edit-distance metrics to compare words.

In the rest of this section, we first describe each of the components of our analysis: alignment of words with UMLS concepts; parsing; and edit-distance computation as well as detection of negations. We then describe the method used to combine these elements into a similarity computation for any pair of phrases.

#### Mapping Phrases to UMLS

UMLS terms can be used to describe a large proportion of the words or entities in the phenotype phrases. A term may be a pathology name like "*obesity*", a modifier such as "*mild*", an anatomy location such as "*wrist*" or a multiple word entity like "talipes *equinovarus*". When we find them, UMLS terms help us recognize multiple word entities and classify terms and words into categories such as pathology, modifier, anatomy or named entity.

We used MetaMap Transfer [9] (version 2.4 with 2006 AA UMLS version using the default parameters) to annotate all the phrases found in the OMIM Clinical Synopsis with UMLS information (we use default settings, this stage is automatic and takes about 12 hours per update). MetaMap produces UMLS annotations and performs two major tasks:

1. It identifies multi-word expressions as single terms. For example "*congenital abnormality*" is tagged as a single token.

2. It classifies each term according to the UMLS controlled vocabulary.

For example, "*strokes due to coagulopathy*" is mapped to "Strokes [CUI 0038454/Disease or Syndrome] and Coagulopathy [CUI 0005779/Disease or Syndrome]".

Not all term classifications computed by MetaMap are correct. Previous work indicates precision as low as 0.69 [12] or 0.56 [13] if no precaution is taken. For example, "*adams stokes attacks*" is mapped to "*adams*" in category: "*Health Care Related Organization*"; in "*functional defects in the cortical and subcortical motor related areas of the frontal lobe*", "*cortical*" is mapped to "*BARK (ADRBK1 gene)*" in category "*Gene or Genome*". Such associations would introduce too much noise in our phrase similarity computation.

Low precision stems from the very large number of different terms in UMLS and the variety of vocabularies combined in UMLS (over 30 distinct vocabularies are merged in the free version of UMLS).

In many applications, the precision of MetaMap is improved by focusing on a subset of the UMLS terms, and defining smaller collections of terms in specific domains within UMLS.

As OMIM phenotypes are extremely varied and cover all domains, such an approach would miss too many of the candidate UMLS concepts. Instead, we rely on UMLS Semantic Categories (such as "Congenital Abnormalities") to filter UMLS concepts returned by MetaMap. We manually defined a set of non-noisy UMLS Semantic Types and keep only concepts that belong to those types in the result of MetaMap. For example, terms that belong to the "Fish","Bird","Organization" semantic types in UMLS are ignored. Those that belong to the categories shown in Table 1 at Additional File 1 are kept.

In our application, we exploit the MetaMap UMLS annotations in terms of "rough semantic" categories. We manually mapped UMLS Semantic Types into groups according to the functional role of the terms in the phenotype descriptions. This reclassification of UMLS concepts into new semantic groups has been suggested by Fan and Friedman [18]. We divided the concepts into 4 semantic roles relevant for OMIM phenotype phrases using the existing semantic classifications of UMLS.. The semantic roles we created attempt to describe the phenotype structure and include the following 4 categories: pathology, anatomy, named entities and modifiers (See Table 2).

**Table 2:** Rough Semantic Categories.

Rough Semantic Category	Description	Examples
Pathology or Finding	Names and symptoms of diseases	"Perthes", "Hexadactyly", "Diffuse atrophy" or "Short finger"

Named entities	Names of chemicals, functions, microorganisms or proteins	"Actin", "Tyrosine", "Insulin" or "Agglutination".

Anatomy	The body part or organ the phenotypes occurs in.	"Cranium bifidum", "Thumb", or "Distal femur"

Modifiers	Concepts describing the phenotype and changing its meaning.	"Absent", "Hypoplastic", "Mild", "Enlarged"

We also use UMLS tagging to reduce the syntactic complexity by recognizing multi-word expressions. We present quantitative evaluation of this simplification in the Results section.

Both the chunking of multiple words into single tokens and their semantic categorization help build a relevant grammar for parsing the phrases in a robust manner.

#### Parsing

The syntactic structure of the phrases is critical to decide on their similarity.

Consider the task of comparing these two phrases: "*fifth finger single interphalangeal crease*" and "*single flexion crease of fifth fingers*". The sub-phrase "*fifth finger*" appears at the beginning of the first and at the end of the second - the edit distance between the two strings would therefore be quite high. However, knowledge of the syntactic structure (NN NN vs. NN of NN) and knowledge that the phrase "*fifth finger*" is identified as a single token of type "Anatomy" can help us compute a more precise similarity between the two phrases.

Because the syntactic structure of phrases in OMIM can be complex (on average 3.9 words with many phrases containing more than 6 words), our approach is to try to parse these expressions, and then compare the parse trees, while taking into account the modification relations that exist between the elements of the phrases. As is well known in natural language processing, parsing noun phrases can be complex because the structure of the noun phrase can be ambiguous. For example, "*altered melatonin secretion*" may be parsed as ("*altered melatonin*" + "*secretion*") or ("*altered*" + "*melatonin secretion*").

Consider the following group of phrases found in OMIM:

• *ossification defect of skull*

• *absent ossification of skull vault*

• *decreased skull ossification*

• *deficient skull ossification*

Our algorithm concluded they all belong to the same cluster of similar phenotype phrases. Wrong parsing of the phrases could have prevented this clustering. For example: (deficient skull ossification) parsed as (deficient skull) (ossification) would not have been similar to (ossification defect) (of skull).

In order to parse Noun Phrases, we must decide on a grammar and a set of categories that annotate words and groups of words within phrases. We use regular parts of speech (verb, adjective, noun, etc) and, in addition to traditional grammar, we introduce as well the UMLS classification of terms into the 4 categories described above: pathology, anatomy, named entity and modifier. If a word or a term is classified by UMLS, we keep its UMLS tag; else we use the parts of speech tagged by a standard parts of speech tagger (we used the LingPipe [19] tagger trained on the Genia corpus [20]).

We then manually constructed a Context Free Grammar (CFG) that captures the structure of the phenotype description in the OMIM Clinical Synopsis. The leaves of the parse trees are tokens annotated either by UMLS categories (pathology, anatomy, named entity or modifier) or by parts of speech tags (conjunction, adverb, etc). Note that as discussed above, the tokens annotated by UMLS can cover multiple words.

We create the tree using the method of chart parsing, gradually building the parse tree by combining simpler parts of the tree together based on the CFG starting from the labels over the phrase in a bottom-up manner (for more information see [21,22]). We used the Chart parser code from AIMA [22]. Phrases longer than 11 words (1,438 such phrases were found out of 68 K) were not parsed as the parser may not finish parsing in a satisfactory time; these phrases are still compared to other phrases using edit distance (computed with dynamic programming) and still affect the similarity measure of the syndromes containing them.

### Negation Detection

Phrases that include negation are not incorporated in any cluster and are not used for computing similarity among syndromes. For example, "*no significant loss of vision" *is not clustered together with "*loss of vision"*, even though they are syntactically similar. Negation is discovered using regular expressions in the same spirit as NegEx [10]. We extend this approach by relying on the structure of the parse tree using the following rules:

1. Phrases with the word "*normal*" appearing anywhere (not as part of another word such as "*abnormal*") are marked as negations. (This is implemented as a regular expression test.)

2. Phrases where the word "*no*" appears are marked as negations only if they contain a "Pathology" node in their parse tree (in order to remove sentences describing absent functions or body parts). For example, "*no dysarthria" *is marked as a negation, because *"dysarthria" *is a pathology but *"no natal teeth*" is not marked as a negation.

#### Clustering of Similar Phenotype Phrases

We compared all pairs of phrases within each area. Altogether, we performed about 77 Million pairwise phrases comparisons (this stage takes about 24 hours using one 3.0 GHZ CPU and is executed each time we update the data from OMIM). We define a set of rules to decide on the similarity of 2 parsed phrases:

Successfully parsed phrases (based on our context free grammar described above) are similar if they share synonymous Pathology/Named Entities and Anatomy node terms and are modified by synonymous modifiers. Nodes annotated by MetaMap are compared by comparing their UMLS tag (CUI). Nodes that are not annotated by MetaMap are compared as case-insensitive string comparison. This comparison relies on the parse tree to determine which parts should be compared (for example: nodes annotated in both trees as "Modifiers" would be compared to each other when comparing the "Pathology" they modify. Both phrases must have a similar "Pathology" node for the rest of the nodes to be compared). Phrases without a parse tree were compared using string distance (allowing maximum distance of 2).

## Results

Our objectives are: create cluster of similar phenotype phrase and identify negation phrases. In this section we evaluate each of the steps in our method: (1) classification of phenotype phrases into areas; (2) MetaMap tagging of the phenotype phrases; (3) parts of speech tagging of the phrases; (4) parsing of the phrases according to our CFG grammar; (5)negation detection; and (6) clustering of the phrases.

### Distribution of phenotype phrases into areas

Our method identifies clusters of similar phenotype phrases (*i.e*., phrases have similar meaning) within each area. We originally found 159 distinct headings and sub-headings in the Clinical Synopsis sections in OMIM. We mapped every phenotype phrase to one or more of the 26 areas on the basis of its heading/sub-heading location in OMIM, syndrome names were gathered in a dedicated area (see Table 2 of the Additional File 1).

Area 26, "Misc", is not used further in clustering experiments. Phrases in this area include: "*variable phenotype" *or "*reduced penetrance*".

This manual classification improved overall the consistency of the rough classification of phenotypes. The granularity we obtain with 25 areas allows us to significantly reduce the complexity of the task of finding synonym phrases: we avoid false positive matches by only comparing phrases within the same area. In addition, the consistent classification in areas improves the precision of UMLS mapping, as has been discussed in the past by Chapman *et al*. [12].

### MetaMap Performance

In the dataset of all Clinical Synopsis phrases in the clustered areas (31,778 distinct phrases out of 67,470 phrases extracted from OMIM.txt), Meta Map tagged 78% of the word tokens and identified on average 1.9 concepts (CUIs) per phrase. MetaMap tagged 41,566 concept instances in the dataset covering 104,673 words. Many of the identified concepts include more than one word (33,591 concept instances of more than one word were identified). The grouping of words into multi-word tokens by MetaMap significantly reduces the syntactic complexity of the phrases: before the MetaMap treatment, phrases have an average length of 3.86 tokens (standard deviation of 2.02); after the MetaMap tagging, phrases have an average length of 2.48 tokens (with standard deviation of 1.4). For example: "*bowed radius*", "*bowing of the radius*", "*bowing of radius*" and "*bowed radii*" are all changed to "*bowing radius*".

We did not measure explicitly the precision of the MetaMap annotations after filtering according to UMLS Semantic Types.

### Parsing Results

**Figure 2:**
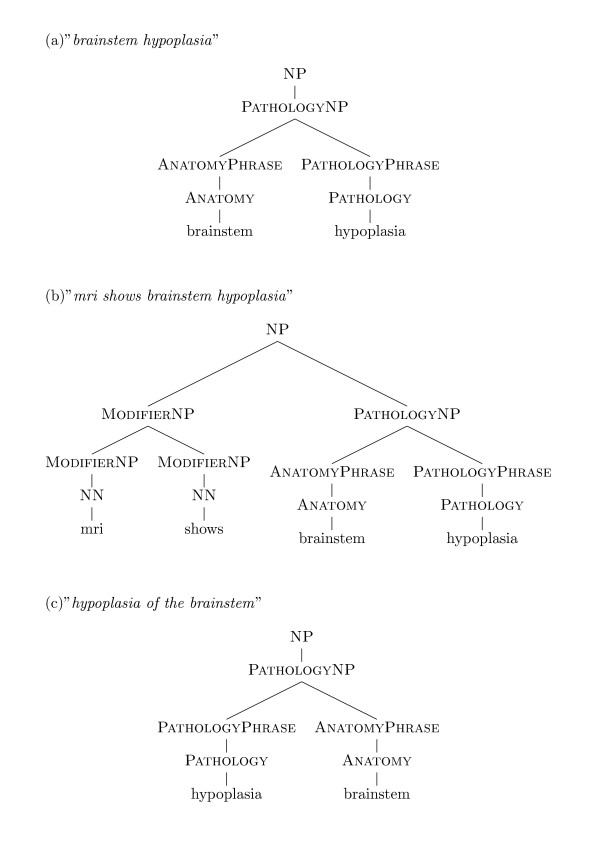
**Parsing result**. a) The phrase "brainstem hypoplasia" parsed. The token "brainstem" was recognized by MetaMap as UMLS term (CUI C0006121) of semantic type "Body Part, Organ, or Organ Component" and the token "hypoplasia" was identified as CUI C0243069 of semantic type "Pathologic Function". b) Parse tree of the phrase "mri shows brainstem hypoplasia", same concepts were recognized as in (a), "mri" is marked as a noun and "shows" is marked as a noun as well since we view the phrases as noun phrases without verbs. c) Parse tree of the phrase "hypoplasia of the brainstem", the entire phrase is reduced by MetaMap to the two concepts identified in (a), only in reversed order.

The parse trees we obtained have an average height of 3.75 and size of 5.3 nodes on average. The parser found an acceptable parse tree (*i.e*., one matching our grammar) in about 90% of the phrases. The percentage of phrases parsed successfully varies between the different areas (84% - 93%). In one area (25 - lab) parsing encountered problems due to the different structure of the phrases which includes many prepositions in the same sentence and a large number of named entities. Figure 2 shows example parse trees that demonstrate the multi-word tokens tagged by MetaMap.

### Negation Detection Results

860 distinct phrases were identified as negation phrases (3.2% of the phrases in the applied areas: 2 - 25). Precision (the fraction of correctly identified negations out of all identified negations), measured by manual inspection of 10% of the negation phrases (chosen randomly and tagged manually as negated or not-negated), was 89% with most false positive phrases being ambiguous for human readers as well ("*low to normal IQ*", "*plasma testosterone is normal or increased*"). Measuring the recall (number of negation phrases identified/number of negation phrases in the database), in the absence of a well annotated examples set, is too difficult.

**Figure 3:**
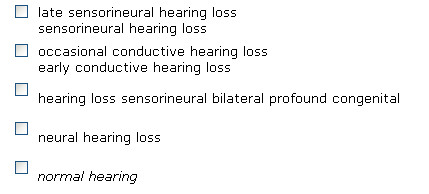
**CSI-OMIM: Negation detection**. Negations are marked with italics and are ignored in the clustering process.

When searching OMIM using our website, negation phrases are marked by italics. These phrases are also not included in the similar phrase clusters ("*loss of vision*" is not grouped with "*no significant loss of vision*" even though both contain the phenotype "*loss of vision*") see Figure 3.

### Clustering of Similar Phenotype Phrases Results

**Figure 4:**
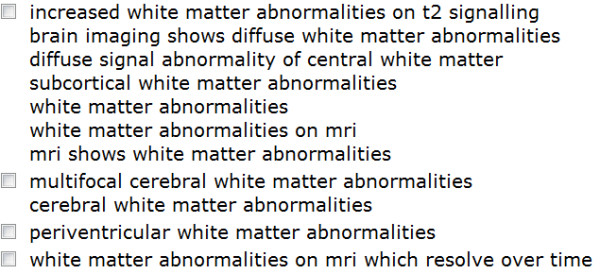
**CSI-OMIM: Clusters of similar phrases**. Clusters of similar phrases results of "white matter abnormalities" search.

Our algorithm groups similar phrases into clusters that are recognized as synonyms (see Figure 4). We obtained altogether 1,680 clusters of similar phenotype phrases covering 4,551 distinct phrases with on average 2.7 phrases per clusters of similar phenotype phrases.

Clusters of similar phenotype phrases cover 13,009 phrase instances (about 20% of the overall phrase instances in the clustered areas). The distribution of clusters per area that we eventually obtained is shown in Table 1.

For example: "*hyperreflexia in the lower limbs*" is clustered with "*hyperreflexia especially of the lower limbs*", for two reasons: a) in the parse tree of both the "Pathology" node is similar b) both nodes are connected to a similar "Anatomy" node.

In Figure 2 we see 3 phrases which are clustered together due to an identical PathlogyNounPhrase sub-tree in the parse tree of phrases (a) and (b), and a PathlogyNounPhrase sub-tree with identical Pathology and Anatomy between (c) and (a).

Precision found in predicted synonymous phrase pairs was 93.5% (one thousand pairs of phrases were sampled randomly from the resulting phrase clusters and evaluated by a geneticist as similar or non-similar). Recall was not measured due to the complexity of the problem and lack of an expert annotated corpus.

The clusters obtained through our method are used in two ways: when a user enters a term that is recognized as a member of a cluster, the system searches for all variants of the term as well. For example, if the user searched for "*thickened cranium*", results that include "*thickened skull vault*" and "*thickened cranial vault*" will also be retrieved.

We measure the quality of the acquired clusters by measuring the improvement they bring to a data mining application. We want to compute a similarity measure between syndromes. To this end, we compare the Clinical Synopsis section of the syndromes, after they have been parsed and normalized - that is, all occurrences of phrases that belongs to the same cluster, are replaced by the same term. For example, the syndrome "CUTIS MARMORATA TELANGIECTATICA CONGENITA" (OMIM 219250) is normalized "*bowed legs*" replaced by the cluster: "*bowing of the legs*; *bowed legs*; *bowing of legs*". We then compute the cosine distance of the normalized syndromes and compare the results when computing the cosine distance without normalization.

Cluster normalization increased the number of similar phenotypes among syndromes:

79,770 new connections between syndromes were discovered among 4,802 syndromes, adding 16 new connections (shared pehnotypes) per syndrome on average. For example: the aforementioned syndrome 219250 is now connected to "BOWING OF LEGS, ANTERIOR, WITH DWARFISM" (OMIM 112350) through the phenotype "*bowing of the legs*" due to the aforementioned cluster.

## Conclusions

Our work focused on pre-processing the textual component of the OMIM dataset using Natural Language Processing methods, and produced clusters of similar phenotype descriptions. These similar clusters allow us to propose an enhanced search system of the OMIM database and to perform effective data-mining on the OMIM textual descriptions and discover similarities among syndromes.

Our work unifies parts of the methods used by Lage *et al*. [3] and Van Driel *et al*. [6] and improves the precision of the results by dividing Clinical Synopsis phrases into areas and using MetaMap to get both UMLS and MESH information. We provide a robust application capable of being updated regularly and offering a wealth of organized phenotypic data of OMIM syndromes.

Precision found in predicted synonymous phrase pairs was 93.5%. 3.2% of the phrases were recognized as negations with a precision of 89%. We identified 79,770 new connections between syndromes - on average 16 new connections per syndrome.

Using the new curated database, we provide an online search application for clinicians with improved search accuracy. The improved data is used to find similarity between syndromes in order to find candidate genes for hereditary syndromes as part of the S2G application [17]. The enhanced OMIM database we produced can be further used for bioinformatics purposes as a basis for identifying connections between syndromes. Another product of our work is an accurate connection of syndromes' phenotypes to the UMLS and MeSH, this can be applied further in the development of literature mining tools and search applications. The database is available in our website (in the FAQ section).

Our system contains over to 31 thousand phenotype phrases in comparison to 9,500 in the Human Phenotype Ontology (HPO) [15,16]. Our method may be used as a way to augment HPO by suggesting synonyms or improve syndrome comparison by including terms which are not yet in the vocabulary but exist in OMIM.

Another aspect of our work is the principle of detecting similarity between phenotype descriptions that can be applied to vocabularies using the semantic roles we defined as anchors for comparison. These rough semantic categories (Pathology or finding, Anatomy or body area, Modifiers and named entities, see Table 2.) can assist in detecting similarity of concepts by identifying important cues in the phrases: pathologies and anatomy region and named entities (in this order), and then comparing the modifiers. This distinction can enhance both statistical driven methods for similarity computation by measuring co-occurrence in documents and construction and improvement of manually built ontologies such as HPO.

Our pre-processing allows finding similarity between syndromes based on different measures of the descriptions using the rough semantic categories. For example similarity can be calculated based only on anatomical location terms or only pathology terms. Syndrome similarity can also be calculated using only a subset of the anatomical areas such as: similarity based only on the "Hair", "Nails" and "Skin" areas.

## Authors' contributions

RC and AG contributed to the design of the study equally. RC carried out the design of the comparison algorithm the implementation and drafting the article. ME contributed in evaluating the results and drafting the article. OB coordinated the study and helped draft the manuscript. All authors read and approved the final manuscript.

## Supplementary Material

Additional file 1 **Supplementary Material**. Details of data acquisition, UMLS division into rough semantic categories and division of heading/sub-headings into areas. Click here for file
