# Supplementary material for: CSI-OMIM - Clinical Synopsis Search in OMIM
Source: BMC Bioinformatics. 2011 Mar 1;12:65. doi: 10.1186/1471-2105-12-65 (PMC3053257; doi:10.1186/1471-2105-12-65)
Supplement: Additional file 1 — Supplementary Material. Details of data acquisition, UMLS division into rough semantic categories and division of heading/sub-headings into areas. [file 1471-2105-12-65-S1.DOC]

# Supplementary Meterial

OMIM data is downloaded from <ftp://ftp.ncbi.nih.gov/repository/OMIM/omim.txt.Z>, omim.txt is extracted from the archive using gunzip.

## Table 1 - UMLS Semantic Types used in our method

| **Category** | **Semantic types** |
| --- | --- |
| Pathology or Finding | Congenital Abnormality; Mental or Behavioral Dysfunction; Disease or Syndrome; Sign or Symptom; Anatomical Abnormality; Neoplastic Process; Acquired Abnormality; Sign or Symptom; Finding; Pathologic Function; Neoplastic Process; Anatomical Abnormality,Intellectual Product; Organ or Tissue Function; Disease or Syndrome; Cell or Molecular Dysfunction; Injury or Poisoning; Environmental Effect of Humans; Mental or Behavioral Dysfunction |
| Named entities | Biologically Active Substance; Lipid; Pharmacologic Substance; Hormone; Neuroreactive Substance or Biogenic Amine; Organic Chemical; Carbohydrate; Enzyme; Amino Acid, Peptide, or Protein; Element, Ion, or Isotope; Hazardous or Poisonous Substance; Immunologic Factor; Occupation or Discipline; Nucleic Acid, Nucleoside, or Nucleotide; Body Substance; Molecular Function; Cell Function; Phenomenon or Process; Daily or Recreational Activity; Idea or Concept,Spatial Concept; Diagnostic Procedure; Organ or Tissue Function; Clinical Attribute; Phenomenon or Process; Bacterium; Laboratory or Test Result; Physiologic Function; Inorganic Chemical; Steroid; Physical Object; Antibiotic; Biomedical or Dental Material; Event |
| Anatomy | Organism Attribute; Spatial Concept; Cell; Tissue; Fully Formed Anatomical Structure; Embryonic Structure; Body Part, Organ, or Organ Component; Body System; Body Location or Region; Body Space or Junction; Functional Concept; Receptor |
| Modifiers | Quantitative Concept; Qualitative Concept; Spatial Concept |

## Table 2 - Division of OMIM Headings, Sub-Headings to areas.

Headings are marked with capital letters.

Disease mechanism such as "Molecular Basis" and "Gene" (For example: "Caused by mutations in the hexosaminidase A" [OMIM 272800]) were disregarded since they describe the cause but not the phenotype of the disease.

| **Area Name** | **Heading and Sub-Heading** |
| --- | --- |
| Syndrome names | N/A |
| Abdomen/gi | GASTROINTESTINAL; ABD; ABDOMEN; ABDOMEN_gastrointestinal; SPLEEN; ABDOMEN_biliary tract; PANCREAS; ABDOMEN_pancreas; LIVER; ABDOMEN_spleen; ABDOMEN_liver; GI; ABDOMEN_external features |
| Respiratory | RESPIRATORY_nasopharynx; LUNGS; RESP; LUNG; PULMONARY; RESPIRATORY_lung; RESPIRATORY; PULM; RESPIRATORY_airways; ULMONARY; RESPIRTORY; RESPIRATORY_larynx; VOICE |
| Gu/renal | GENITOURINARY_ureters; GENITOURINARY_bladder; GENITOURINARY_kidney; GENITOURINARY_kidneys; RENAL; GENITOURINARY; GU |
| Gu/genitalia | GENITOURINARY; GENITOURINARY_external genitalia; female; GENITOURINARY_external genitalia; male; GENITOURINARY_internal genitalia; male; GENITOURINARY_internal genitalia; female; GU |
| Cardiovascular | VASCULAR; CARDIOBASCULAR_heart; CARDIOVASCULAR; CARDIOVASCULAR_cardiac; CARDIOVASCULAR_heart; CARDIAC; CARDIOVASCULAR_vascular |
| Muscle | MUSCLE; SOFT TISSUE; USCLE; MUSCLES; USCELE; MUSCLE |
| Endo | ENDOCRINE; ENDOCRINE FEATURES; ENDO |
| Neuro | DEVELOPMENT; NEUROLOGIC_behavioral/psychiatric manifestations; NEUROLOGIC; NEURO; NEUROLOGIC_central nervous system; NEUROLOGIC_peripheral nervous system; EURO; BRAIN; HEAD; HEAD AND NECK_head; HEENT; SPINE |
| Oncology | NEOPLASIA; NEOPLASM; ONCOLOGY; NCOLOGY; HEME; HEMATOLOGY |
| Heme | HEME; HEMATOLOGY; LYMPHATICS |
| Immune | IMUNOLOGY; IMMUNE; IMMUNOL; IMMUNOLOGY; IMMUNOGLOBULIN; IMMUNOLOGIC |
| Eyes | HEAD_eyes; EYES; HEAD AND NECK_eyes; YES; EYE; HEAD AND NECK_eye; HEENT; EENT |
| Face | FACE; HEENT; HEAD AND NECK_face; FACIES; MANDIBLE; TONGUE; MOUTH; EAD AND NECK_mouth; EENT_HEAD AND NECK_pharynx; HEAD AND NECK_nose; NOSE; HEAD AND NECK_ears; EARS; EAR; HEAD; HEAD AND NECK |
| Teeth | TEETH; HEAD AND NECK_teeth; HEENT; HEAD AND NECK |
| Neck | NECK; HEAD AND NECK_neck; HEENT; HEAD AND NECK; EENT |
| Head | CRANIUM; HEAD; HEAD AND NECK_head; SKULL; SKELETAL_skull; HEAD AND NECK; HEENT_SKELETAL; SKEL; EENT |
| Limb | SKELETAL_limbs; LIMB; SKELETAL_hand; SKELETAL_hands; SKELETAL_feet; LIMBS; SKELETAL_foot; SKELETAL; SKEL; RADIOLOGY; JOINT; JOUNTS; JOINTS; OINTS; HIPS |
| Skel | SKELETAL; SKEL; SKELETAL_pelvis; SPINE; SKELETAL_spine; RADIOLOGY; HIPS |
| Chest | CHEST_diaphragm; THORAX; CHEST_breasts; CHEST_external features; CHEST; CHEST_ribs; sternum; clavicles; and scapulae; CHEST_ribs and sternum; SKELETAL; SKEL; RADIOLOGY; THYMUS |
| Growth | GROWTH_height; GROWTH; GROWTH_other; GROWTH_weight |
| Nails | NAILS; SKIN, NAILS, HAIR_nails; SKIN, HAIR, NAILS_nails; SKIN, NAILS AND HAIR; SKIN, NAILS, HAIR |
| Skin | SKIN, NAIL, HAIR_skin; SKIL, NAILS, HAIR_skin; SKIN, NAILS, HAIR_skin; KIN; SKIN, NAILS AND HAIR; SKIN; SKIN, NAILS, HAIR; SKIN, NAILS AND HAIR_skin; SKIN, HAIR, NAILS_skin |
| Hair | SKIN, HAIR, NAILS_hair; SKIN, NAILS, HAIR_hair; HAIR; SCALP; SKIN, NAILS AND HAIR; SKIN, NAILS, HAIR |
| Lab | METABOLISM; METABOLIC; METABOLIC FEATURES; LAB; LABORATORY ABNORMALITIES; DIAGNOSTIC LABORATORY; LABORATORY |
| Misc | MICSELLANEOUS; PRENATAL MANIFESTATIONS_movement; MISCELLANEOUS; PRENATAL MANIFESTATIONS; PRENATAL MANIFESTATIONS_delivery; MISC; PRENATAL MANIFESTATIONS_amniotic fluid; PRENATAL MANIFESTATIONS_maternal; PRENATAL MANIFESTATIONS_placenta and umbilical cord; RENATAL MANIFESTATIONS_placenta & umbilical cord |
